# Electroretinographic oscillatory potentials in Leber hereditary optic neuropathy

**DOI:** 10.1007/s10633-024-09968-9

**Published:** 2024-03-07

**Authors:** Mirella T. S. Barboni, Maja Sustar Habjan, Sanja Petrovic Pajic, Marko Hawlina

**Affiliations:** 1 Department of Ophthalmology, Semmelweis University, Budapest, Hungary; 2 Eye Hospital, University Medical Centre Ljubljana, Grablovičeva 46, 1000 Ljubljana, Slovenia; 3 Clinic for Eye Diseases, University Clinical Center of Serbia, Belgrade, Serbia; 4 Medical Faculty, Department of Ophthalmology, University of Ljubljana, Grablovičeva 46, 1000 Ljubljana, Slovenia

**Keywords:** LHON, Retina, Electroretinogram, Photopic negative response, Oscillatory potentials

## Abstract

**Purpose:**

Leber hereditary optic neuropathy (LHON) affects retinal ganglion cells causing severe vision loss. Pattern electroretinogram and photopic negative response (PhNR) of the light-adapted (LA) full-field electroretinogram (ERG) are typically affected in LHON. In the present study, we evaluated dark-adapted (DA) and LA oscillatory potentials (OPs) of the flash ERG in genetically characterized LHON patients to dissociate slow from fast components of the response.

**Methods:**

Seven adult patients (mean age = 28.4 ± 5.6) in whom genetic diagnosis confirmed LHON with mtDNA or nuclear DNAJC30 (arLHON) pathogenic variants were compared to 12 healthy volunteers (mean age = 35.0 ± 12.1). Full-field ERGs were recorded from both eyes. Offline digital filters at 50, 75 and 100 Hz low cutoff frequencies were applied to isolate high-frequency components from the original ERG signals.

**Results:**

ERG *a*-waves and *b*-waves were comparable between LHON patients and controls, while PhNR was significantly reduced (*p* = 0.009) in LHON patients compared to controls, as expected. OPs derived from DA signals (75 Hz low cutoff frequency) showed reduced peak amplitude for OP2 (*p* = 0.019). LA OP differences between LHON and controls became significant (OP2: *p* = 0.047, OP3: *p* = 0.039 and OP4: *p* = 0.013) when the 100 Hz low-cutoff frequency filter was applied.

**Conclusions:**

Reduced OPs in LHON patients may represent disturbed neuronal interactions in the inner retina with preserved photoreceptoral (*a*-wave) to bipolar cell (*b*-wave) activation. Reduced DA OP2 and high-cutoff LA OP alterations may be further explored as functional measures to characterize LHON status and progression.

## Introduction

Leber hereditary optic neuropathy (LHON) is a rare inherited disease predominantly affecting males with a prevalence in Europe of approximately 1:45,000 considering the three primary (m.11778G>A, m.14484T>C and m.3460G>A) maternally transmitted mitochondrial DNA (mtDNA) mutations [1]. These mtDNA mutations are associated with abnormal cellular respiratory chain function causing oxidative stress [2] that severely affects retinal ganglion cells (RGCs) causing progressive optic atrophy [3–6]. The changes in RGCs and in the retinal nerve fiber layer (RNFL) strongly impact the central retina leading to low vision or irreversible legal blindness with small number of patients that eventually recover vision partially [7, 8]. Equivalent mitochondrial alterations can be also caused by biallelic mutations in genes of the nuclear DNA leading to an autosomal recessive form of LHON, as recently reported [9, 10].

There are no specific signs of retinal alterations preceding the conversion to the acute phase in LHON patients [11]. The discovery of retinal biomarkers preceding the conversion could be relevant for early interventions. In addition to the structural examination with optical coherence tomography (OCT), visual electrophysiology plays an important role in the assessment of retinal integrity in LHON. Typically, affected LHON patients show abnormal pattern-reversal visual evoked potential (VEP) and pattern electroretinogram (PERG) with abnormal N95 amplitude or N95/P50 amplitude ratio and shortening of P50 peak time, revealing the primary dysfunction of RGCs [12–17]. The *a*-wave and the *b*-wave of the standard (ISCEV) full-field flash ERG may be classified as normal or slightly reduced in LHON patients [16, 18]. On the other hand, the photopic negative response (PhNR) originating in the inner retina, dependent on RGCs’ integrity [19], has been described as altered and associated to the disease progression in LHON patients [17, 18, 20].

In addition to the PhNR, oscillatory potentials (OPs) have been long reported to originate in the inner part of the retina, reflecting inhibitory/excitatory interactions involving bipolar and amacrine cells in the inner plexiform layer of the primate retina [21, 22]. Each individual OP wavelet may be originated by a different subset of cells which may provide the possibility of accessing specific retinal mechanisms by evaluating individual and consecutive OP peaks [23–26]. Considering the proximity and physiological interdependence of RGCs and the OP generators, it could be speculated that LHON patients are at higher risk of presenting OP dysfunctions. Reduction in OP amplitudes were reported in patients with dominant optic atrophy with OPA1 gene mutations [27] and glaucoma [28, 29], conditions predominantly affecting the retinal ganglion cells. We are not aware of any prior study investigating OPs in LHON.

In the present study, OPs were extracted from DA and LA full-field ERG signals with the application of digital filters using different cutoff frequencies in genetically confirmed LHON patients to evaluate whether OP changes reflect retinal alterations caused by LHON.

## Methods

### Participants

Participants were 7 young adult patients aged 20 to 34 (mean age = 28.4 ± 5.6, 5 males) and 12 healthy volunteers (mean age = 35.0 ± 12.1, 2 males). All subjects underwent complete ophthalmological examination including spectral-domain optical coherence tomography (SD-OCT). All patients showed typical LHON phenotype in chronic phase: bilateral low visual acuity; presence of central scotoma in the visual field; pale (atrophic) optic disk; retinal atrophy with predominant thinning of the ganglion cell complex both in macular and peripapillary areas; and PERG and VEP findings typical for optic neuropathies. All patients were genetically tested for mtDNA and clinical exome. Table 1 shows that six out of seven patients showed pathogenic mtDNA mutations, while one patient (number 7) showed autosomal recessive DNAJC30 152 A > G nuclear DNA mutation. One eye of the controls and LHON patients (bold VA values) were selected for statistical comparisons. Only results of the right eyes were considered, except for patient 4, who in addition to optic nerve atrophy had strabismic amblyopia on the right eye, and patient 6 who showed spontaneous visual recovery of the right eye after several months experiencing blindness (Table 1).

**Table 1:** Participants’ information

P	Age	Sex	LogMAR VA OD	LogMAR VA OS	Gene variant
1	34	M	**2.3**	2.3	m.13042G>T
2	23	M	**2.0**	1.8	m.13042G>T
3	34	F	**1.1**	2.3	m.11778G>A
4	20	M	1.7	**1.7**	m.3700G>A
5	33	M	**1.3**	1.3	m.3460G>A
6	28	M	0.0	**1.0**	m.14484T>C
7	27	M	**0.2**	0.2	DNAJC30 152A>G

### ERG recording

Full-field ERGs were recorded using the RetiPort system (Roland Consult, Brandenburg, Germany) from both eyes following the International Society for Clinical Electrophysiology of Vision (ISCEV) Standards [30]. First, pupils were dilated with 1% tropicamide (Mydriacyl, Alcon). ERGs were recorded with HK-loop electrodes [31]. Participants were dark-adapted for 20 min. Full-field stimulus of 20 consecutive flashes of 3.0 cd·s/m^2^ with a 10-s interstimulus interval were delivered for recording DA 3 ERGs. Subsequently, the participants underwent light adaptation to a background of 30 cd/m^2^ for 10-min. Then LA ERGs to 3.0 cd·s/m^2^ flashes were recorded (LA 3 ERG). Signals were amplified with a band-pass filter from 1 to 300 Hz and sampled at 512 plot points within a 150 ms time window, which gives a sampling frequency of 3413.3 Hz. All stimulus and recording conditions followed ISCEV Standard for clinical full-field electroretinography [30], except the lower corner frequency of the amplifier was higher than the recommended (1 Hz instead of 0.3 Hz). ERG signals obtained from 60 consecutive flashes for LA 3 were averaged resulting in a 150-ms epoch. Raw data were exported from the RetiPort system in time–amplitude matrix and analyzed offline.

### Offline signal processing and data analysis

ERG components were analyzed by peak/trough detection: *a*-waves, *b*-waves and photopic negative response (PhNR). The amplitude of the *a*-wave was defined as the difference in microvolts (µV) between the baseline and the minimum value after stimulus onset. The amplitude of the *b*-wave was the difference in µV between *a*-wave trough and the peak of the *b*-wave. PhNR was defined as the difference between the baseline and the late negative component after the *i*-wave, the positive waveform following the *b*-wave. Peak times corresponded to the intervals, in milliseconds (ms), between the stimulus onset and the peak amplitudes.

Isolated oscillatory potentials (OPs) were extracted from the original ERG signals using fast Fourier transform (FFT) and inverse fast Fourier transform (IFFT) MATLAB® (The MathWorks Inc., Natick, Massachusetts, USA) routines. The low-frequency part of the spectrum identified with the FFT was excluded before the application of IFFT. The high cutoff frequency of the band-pass filters was 300 Hz. The low cutoff frequency was 75 Hz for DA signals, following ISCEV recommendations [30], and two cutoff frequencies, 50 Hz and 100 Hz, for LA signals to obtain OPs, respectively, more or less influenced by low-frequency ERG components. While OP2 and OP4 extracted from LA signals with the low-cutoff frequency (50 Hz) filter were always present, the other three major OPs were not always observed. Group comparisons between controls and LHON patients were performed using one-way ANOVA (independent samples t-test) or two-way ANOVA plus Bonferroni post hoc analyses in the presence of within-subjects repeated measurements such as individual OPs. Corrected *p* values < 0.05 were considered statistically significant.

## Results

### Slow DA 3 and LA 3 ERG components

**Fig. 1:**
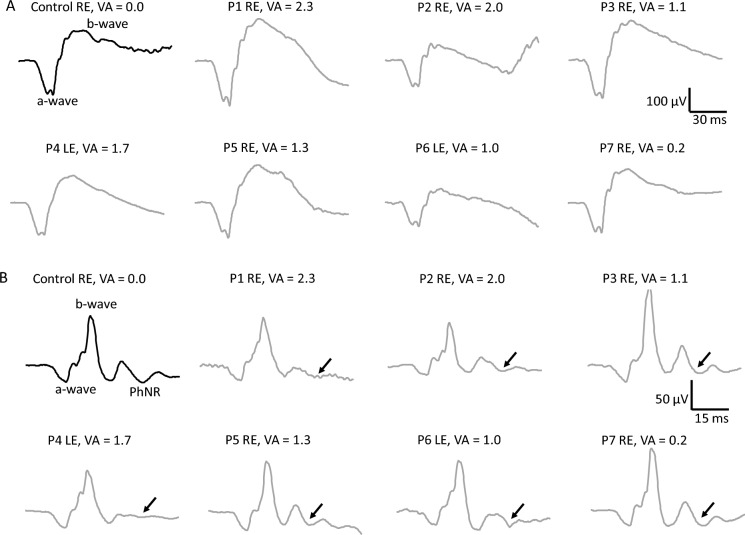
Dark-adapted 3 and light-adapted 3 ERG original responses. Black traces = individual response of a representative subject from the control group with normal VA = 0.0 LogMAR. Gray traces = individual responses from the seven LHON patients included in the study. **A** = dark-adapted responses and **B** = light-adapted responses. Above the signals the eye included as well as the respective VA are shown for all subjects. Note that PhNRs in LHON patients, pointed with the arrows, were reduced in comparison to controls

**Fig. 2:**
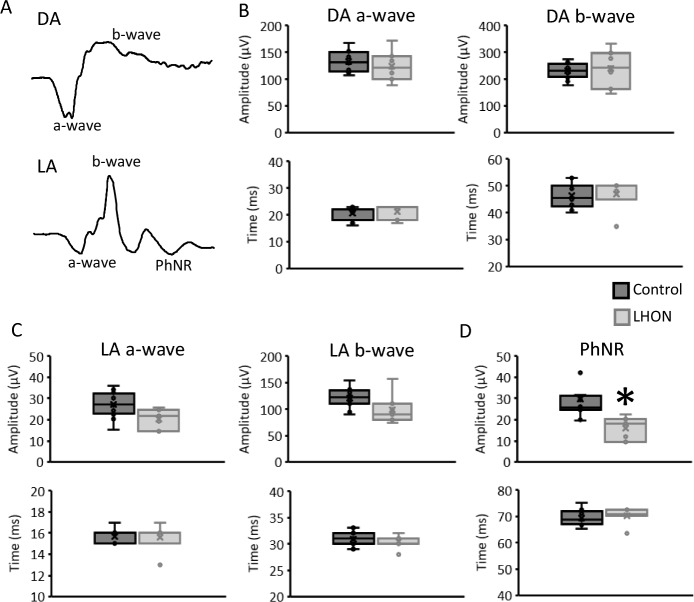
Dark-adapted 3 and light-adapted 3 ERG slow components. Representative control signals for dark-adapted (DA) and light-adapted (LA) ERG responses showing the components analyzed (**A**). Mean/median (box = IQR; whisker = minimum and maximum values) amplitudes (upper boxplots) and peak times (bottom boxplots) for *a*-wave and *b*-wave of the DA 3 ERG (**B**) and LA 3 ERG (**C**), and for the PhNR (**D**). Black symbols = controls. Gray symbols = LHON patients. *Significant difference (*p* < 0.05). PhNR was the only slow ERG component found to be significantly reduced in LHON patients compared to controls

Negative (*a*-wave and photopic negative response: PhNR) and positive (*b*-wave) components of the DA 3 ERG (Fig. 1A) and of the LA 3 ERG (Fig. 1B) were first evaluated to check the integrity of retinal networks generating the low-frequency ERG components. Mean DA and LA control traces including the low ERG components analyzed are shown in Fig. 2A, and the mean (± standard deviation) amplitude and peak time values are shown in Table 2. Figure 2B shows that the mean amplitudes of the DA ERG *a*-wave (*F*_(1,18)_ = 0.736; *p* = 0.403) and *b*-wave (*F*_(1,18)_ = 0.116; *p* = 0.738) were comparable between the groups. Mean peak times of the DA 3 ERG *a*-wave (*F*_(1,18)_ = 0.193; *p* = 0.666) and *b*-wave (*F*_(1,18)_ = 0.157; *p* = 0.697) were also comparable between CTRL and LHON patients. The *b*-to-*a*-wave mean amplitude ratio was slightly larger in LHON patients compared to controls (CTRL = 1.74 ± 0.17 and LHON = 1.92 ± 0.21). However, the difference was not statistically significant (*F*_(1,18)_ = 0.193; *p* = 0.081). LA ERG (Fig. 2C), on the other hand, was not completely comparable between the groups. The *a*-wave (*F*_(1,18)_ = 3.864; *p* = 0.067) and the *b*-wave (*F*_(1,18)_ = 4.290; *p* = 0.054) mean amplitudes showed marginal (nonsignificant) differences between CTRL and LHON groups. The mean peak times of the *a*-wave (*F*_(1,18)_ = 0.047; *p* = 0.830) and the *b*-wave (*F*_(1,18)_ = 0.585; *p* = 0.455) were similar for the LA slow components. In contrast, the mean PhNR amplitude (Fig. 2D) in LHON patients (mean = 15.6 ± 1.3 µV) was about half of the mean PhNR control amplitude (mean = 29.8 ± 11.5 µV). Group comparison showed significantly reduced (*F*_(1,18)_ = 8.870; *p* = 0.009) PhNR amplitudes in LHON patients compared to the control group.

**Table 2:** Comparison of control and LHON patients mean values

	CTRL	LHON	*p*-values
*DA ERG component*			
*a*-wave amplitude (µV)	133.8 ± 18.9	124.6 ± 27.8	0.403
*a*-wave peak time (ms)	20.8 ± 2.5	21.3 ± 2.6	0.666
*b*-wave amplitude (µV)	230.8 ± 29.4	241.3 ± 68.3	0.738
*b*-wave peak time (ms)	46.3 ± 4.6	46.7 ± 5.6	0.697
*b*-to-*a*-wave ratio	1.74 ± 0.17	1.92 ± 0.21	0.081
*LA ERG component*			
*a*-wave amplitude (µV)	27.2 ± 6.1	20.5 ± 4.6	0.067
*a*-wave peak time (ms)	15.7 ± 0.7	15.6 ± 1.3	0.830
*b*-wave amplitude (µV)	121.1 ± 18.1	99.2 ± 28.4	0.054
*b*-wave peak time (ms)	30.9 ± 1.4	30.4 ± 1.3	0.455
PhNR amplitude (µV)	29.8 ± 11.5	15.8 ± 5.5	0.009*

### Dark-adapted OPs in LHON

Five OPs (OP1–OP5) of the DA 3 ERG were analyzed in the time domain by peak/trough detection after the application of an offline band-pass 75–300 Hz filter. Additional filtering with different band-pass filters did not show any significant changes between the groups. Therefore only results of ISCEV standard low cutoff frequency of 75 Hz are reported.

**Fig. 3:**
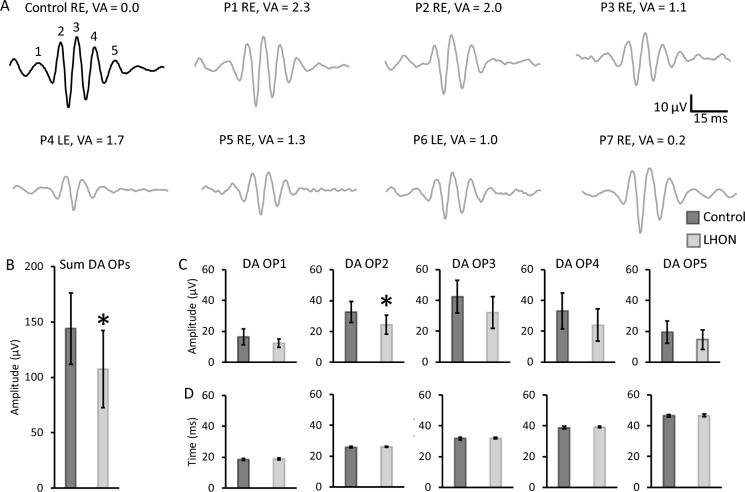
Oscillatory potentials of the DA 3 ERG. DA OP traces of a representative control subject and all LHON patients examined with the respective eye tested and LogMAR visual acuity (**A**). DA OPs (OP1–OP5) were derived from DA ERG signals with 75–300 Hz band-pass digital filter. Mean (± one standard deviation) control (black) and LHON patients (gray) sum OP amplitudes (**B**) and amplitudes (upper plots, **C**) and implicit times (bottom plots, **D**) of controls (black) and LHON patients (gray). *Significant difference (*p* < 0.05)

Figure 3A shows the OP trace of a representative subject of the control group and individual OP traces from the LHON patients showing that the five OPs were detected in all traces. Although completely preserved dark-adapted *a*-waves and *b*-waves were found in LHON patients, as described in the previous section (Figs. 1 and 2), dark-adapted OPs were reduced in LHON patients. Analysis of variance showed that the sum OP amplitude was just slightly attenuated (*F*_(1,18)_ = 5.397, *p* = 0.033) in LHON patients compared to controls (Fig. 3B). However, when individual OP amplitudes were compared between the groups, OP2 (Fig. 3C) was found to mainly drive this group difference (*F*_(1,18)_ = 6.987, *p* = 0.017) while other OPs were just slightly reduced (Fig. 3D) and statistically comparable between the groups (OP1: *F*_(1,18)_ = 3.841, *p* = 0.067; OP3: *F*_(1,18)_ = 4.263, *p* = 0.055; OP4: *F*_(1,18)_ = 2.851, *p* = 0.110; OP5: *F*_(1,18)_ = 2.109, *p* = 0.165). Figure 3E shows that OP peak times were all comparable between the groups (*F*_(1,18)_ < 1.5, *p* > 0.25).

### Light-adapted OPs in LHON

**Fig. 4:**
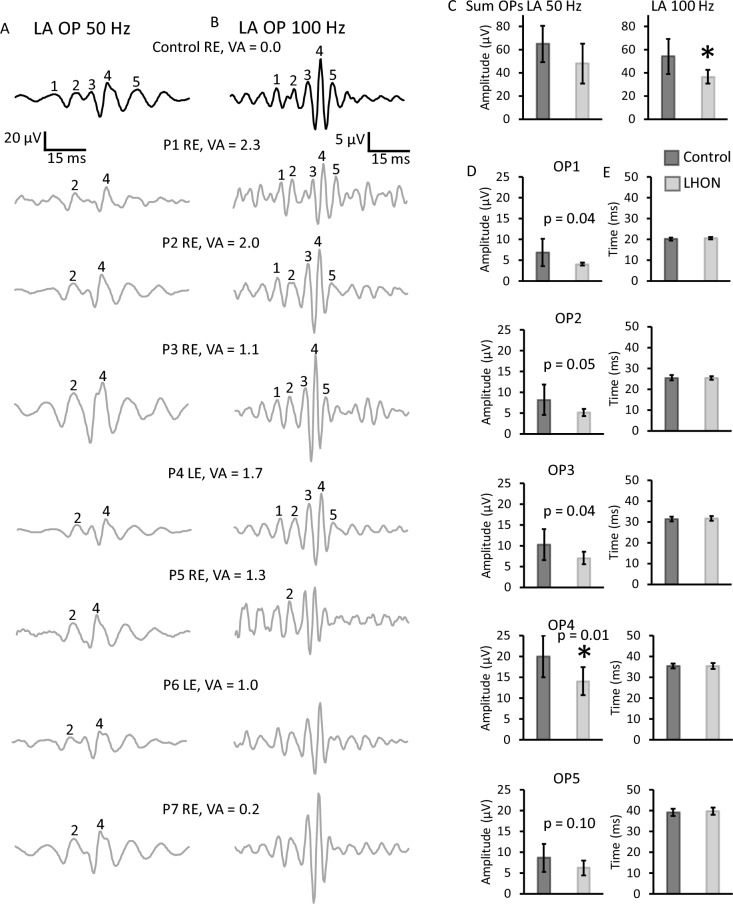
Oscillatory potentials of the LA 3 ERG. LA OP traces of a representative control subject and all LHON patients examined with the respective eye tested and visual acuity (**A**, **B**). OPs were derived from LA ERG signals with 50–300 (**A**) and 100–300 (**B**) Hz band-pass digital filters. Mean (± one standard deviation) control (black) and LHON patients (gray) sum OP amplitudes (**C**). Mean (± one standard deviation) amplitudes (left plots, **D**) and implicit times (right plots, **E**) of controls (black) and LHON patients (gray) for the 100 Hz filter. *Significant difference (*p* < 0.01). Sum OPs as well as individual OPs differences were more pronounced in the 100 Hz filter condition

LA 3 ERG signals (Fig. 1B) were filtered using two offline band-pass filters: one was set at 50 Hz low cutoff frequency and the other was set at 100 Hz low cutoff frequency. In both conditions 300 Hz was selected as the high cutoff frequency. The OP signals extracted from the original LA 3 ERG are shown in Fig. 4 for a representative subject of the control group and the individual OP traces from LHON patients. In both filter conditions five OPs (OP1–OP5) were observed. However, for the 50 Hz condition, OP1 was near the baseline level and OP3 was not always measurable. OP5 implicit time was usually over *b*-wave peak and, therefore, strongly influenced by the PhNR. Therefore, only major components, OP2 and OP4, were analyzed in the time domain using 50 Hz cutoff filter. OP4, the largest oscillation, coincided in time with the *b*-wave peak (control mean *b*-wave peak time = 30.9 ± 1.4 ms and control OP4 peak time = 30.6 ± 1.3), as expected. Therefore, it was more influenced by the *b*-wave peak. At 100 Hz cutoff condition, LA OP waveforms were less influence by the slow ERG components. All five OPs were measurable in all subjects and they were analyzed in the time domain by peak/trough detection, as shown in Fig. 4B. At 50 Hz filter condition, sum OPs (OP2 amplitude + OP4 amplitude) were slightly reduced in LHON patients (Fig. 4C), but still comparable to controls (group effect: *F*_(1,18)_ = 4.731 and *p* = 0.050): mean control = 65.1 ± 15.8 and mean LHON = 48.2 ± 17.2 µV. The within-subjects variable, using two-way analysis of variance, revealed a marginal OP*group effect (*F*_(1,18)_ = 4.377 and *p* = 0.052) with significant group difference for OP4 amplitude (*F*_(1,18)_ = 5.463 and *p* = 0.032) but not for OP2 amplitude (*F*_(1,18)_ = 2.856 and *p* = 0.109).

On the other hand, sum OP (Figure 4C), group effects of the LA OPs extracted with 100 Hz cutoff frequency showed more evident group differences (*F*_(1,18) _= 8.330 and *p *= 0.010) when comparing controls (mean = 54.2 ± 15.3) with LHON patients (mean  =  36.6 ± 5.8). Further analysis including OP as within-subjects variable using two-way analysis of variance revealed no significant OP*group effect (*F*_(1,18) _= 1.634 and *p *= 0.176). However, Figure 4D shows that individual OP amplitudes were significantly affected in LHON patients. While early OPs showed only marginal differences (*F*_(1,18) _< 5.198 and *p *> 0.036), OP4 amplitude reduction was statistically significant (*F*_(1,18) _= 7.798 and *p *= 0.013). OP5 amplitude was comparable between the groups (*F*_(1,18) _= 2.986 and *p *= 0.102). The comparisons of OP peak times (Figure 4D right column) showed no significant group (*F*_(1,18) _= 0.139 and *p *= 0.714) or OP*group (*F*_(1,18) _= 0.194 and *p *= 0.665) effects. The individual analysis also revealed no group effects on OP peak times (*F*_(1,18) _< 0.902 and *p *> 0.356). Finally, there were no correlations between PhNR and LA OP amplitudes (Spearman correlation = − 0.17 to 0.33 and *p *> 0.4).

## Discussion

In addition to the well-known, and confirmed by this study, inner retina dysfunction reflected by pattern ERG (PERG) and photopic negative response (PhNR) changes, the present report shows that oscillatory potentials (OPs) are also affected in LHON patients. Retinal alterations caused by LHON were reflected in full-field dark-adapted (DA) and light-adapted (LA) ERG OPs. Interestingly, LA OP abnormalities were more evident when slow components were filtered out using a higher-band-pass digital filter. The present data also confirm preserved or relatively preserved photoreceptor to bipolar cell (outer retina) mechanisms as revealed by normal *a*-wave and *b*-wave values in the DA responses (Fig. 1A) and close to normal values in the LA responses (Fig. 1B) in our cohort, as previously reported [17, 18, 20]. In addition, LHON patients also showed affected inner retinal components of the multifocal ERG [32].

The OPs of the full-field flash ERG are low-voltage high-frequency electric oscillations consistently observed in the rising phase of the *b*-wave [26, 33–35]. DA OP analysis is recommended by ISCEV [30] using band-pass (~ 75–300 Hz) filter of signals obtained with the standard (3.0 cd·s/m^2^) DA full-field ERG. Interestingly, individual OPs are generated by distinct retinal mechanisms which are also differentially influenced by the flash intensity and the state of adaptation [24–26, 35–37]. It has been proposed that the early OPs may have more distal retinal origins while intermediate and late OPs show inner retinal spiking generators [38]. Since the late OPs are believed to originate from the inner retina (amacrine/ganglion cells) which is severely affected in LHON, we investigated the integrity of the five major DA and LA OPs. DA OPs have been consistently reported to be sensitive to retinal changes caused by diabetes [39–41] and other conditions affecting inner retinal mechanisms [24]. OP alterations may accompany other optic neuropathies as they have been reported in patients with autosomal dominant optic atrophy [27]. However, OPs were not specifically studied in LHON patients. Abnormal OPs have also been reported in patients with glaucoma primarily affecting retinal ganglion cells. These findings suggested amacrine cells alterations in addition to ganglion cells alteration [28, 29]. DA OPs in non-human primate with experimental glaucoma were not consistently different from control eyes [42], while LA OPs were not specifically studied.

In the present report, DA OP differences between controls and LHON patients with completely intact DA *a*-wave and *b*-wave were slight for the sum of the DA OPs (Fig. 3B). The comparisons of individual OPs revealed that OP2 was mainly affected. The LA OPs seemed more affected in LHON patients. Importantly, significant differences between LHON patients and controls for OPs extracted with the 100 Hz low-cutoff frequency filter suggested that fast OPs (105–215 Hz) [23] likely originated at inner retinal cells [38] might be involved in the pathogenies.

There are no current standard recommendations to derive OPs from LA ERG signals although they are clinically relevant. For instance, reduced or absent LA OP2 has been observed in inherited retinal conditions affecting signal transmission between photoreceptors and bipolar cells [43]. Interestingly, in patients with congenital stationary night blindness (CSNB), LA OP2 and OP3 were absent while OP4 was preserved [44]. Likely, abnormal OP2 and OP3 was probably due to a defect in the on-bipolar cells, with normal OP4 as off-bipolar cells were properly functioning. The present data shows asymmetric LA OP alterations in LHON patients with OP4 more prominently reduced. Although the origins of the ERG OPs have been debated for several decades [22, 25, 45, 46], early observations indicated that they may originate in the retinal interneurons with different OPs representing the electrical manifestation of a distinct retinal event [35]. Possibly, the lower OP amplitudes found in LHON patients represent functional changes of the amacrine cells, similar to what has been reported in patients with autosomal dominant optic atrophy [27]. However, a direct effect of RGCs dysfunction on the OP amplitudes [45] may also be taken into consideration when analyzing OP changes in LHON patients.

Visual processing driven by the RGCs that are responsible for sharp vision and color discrimination are at high risk of suffering from mitochondrial dysfunction [47] which may influence inner retinal mechanisms. DA OPs have been long reported to be specifically affected in diabetic patients with no detectable signs of diabetic retinopathy [48], with lower amplitudes of the early OPs correlated with the vascular changes [49]. Microvascular changes have also been considered a pathogenic mechanism and a potential biomarker in LHON patients [50]. Special microvascular properties are present in the optic nerve head that is affected in LHON [5] and may therefore influence DA OP2 amplitudes perhaps in presymptomatic stages. However, other mechanisms affecting the synaptic conduction of affected ganglion cells may play a role in the chronic stage as observed in the patients included in this study.

The limitations of the study were small sample size and the genetic / clinical heterogeneity of LHON patients included in the study. In order to confirm whether this group can be representative of the LHON disease, future studies may consider investigating genetically homogeneous groups as well as subjects with and without microvascular changes (OCT) in presymptomatic stages.

## Conclusion

The particular group of genetically characterized LHON patients in chronic stage of the disease displayed OP abnormalities suggesting inner retinal dysfunction in addition to ganglion cell loss. Inner retinal ERG components such as P50/N95 (PERG) and the PhNR may be affected as a consequence of retinal ganglion cells’ dysfunction. OP abnormalities could be the result of a direct dysfunction of retinal mechanisms other than RGCs. These findings, however, remain to be further explored in terms of pathophysiology and possible cellular generators. Finally, if future investigations show that the OPs are reduced early in the disease process, then it would prove to be a useful biomarker for the diseases progression.

## Declarations

### Statement on the welfare of animals

This article does not contain any animal experiment.
